# Attribution of the Tibetan Plateau to northern drought

**DOI:** 10.1093/nsr/nwz191

**Published:** 2019-11-21

**Authors:** Yuzhi Liu, Yaohui Li, Jianping Huang, Qingzhe Zhu, Shanshan Wang

**Affiliations:** 1 Key Laboratory for Semi-Arid Climate Change of the Ministry of Education, Lanzhou University, China; 2 Institute of Arid Meteorology, China Meteorology Administration, China

The Tibetan Plateau (TP), which is located in Asia and has an average elevation of over 4000 m, acts as a raised source of heat [1] and an isolated region of humidity in the atmosphere [2]. The TP serves as a ‘world water tower’ because it stores large amounts of water as glaciers, lakes and rivers [2]. Furthermore, previous studies have found that the eastward outflow of water vapor and clouds away from the TP contributes significantly to precipitation over downstream regions. However, the dynamic mechanism behind these observations is still unclear. It is known that the key driver in the transportation of air and water resources from the TP is the wind field. Under global warming, the poleward limit change of the tropical Hadley circulation and the thermal effect of the terrain over the TP forces the mid-latitude subtropical westerly jet (SWJ) to shift [3]. However, the true effects of the SWJ shift are unclear. Therefore, to reduce uncertainties in weather forecasting and climate prediction, a dynamic explanation of the relationship between atmospheric circulation and its influence on precipitation over the northern area of China is urgent.

Here, we propose a dynamic mechanism of the northern drought attributable to the TP in summer. The TP, similar to a very large engine, drives the nearby movement of water vapor, clouds and aerosols. This ‘strengthening effect’ controls precipitation near the TP and can trigger flooding or droughts in downstream regions. The northern drought is driven by the collocation of the SWJ position and the TP strengthening effect. The meridional shift in the SWJ is the determining factor of the northern drought in summer. When the SWJ shifts northward, the upper-level westerly wind is weakened; thus, the water vapor, clouds or dusty clouds over the TP are transported northward less often, reducing precipitation and causing more frequent droughts. In contrast, when the SWJ shifts southward, the northern area of China experiences increased precipitation in the summer.

**Figure 1.**
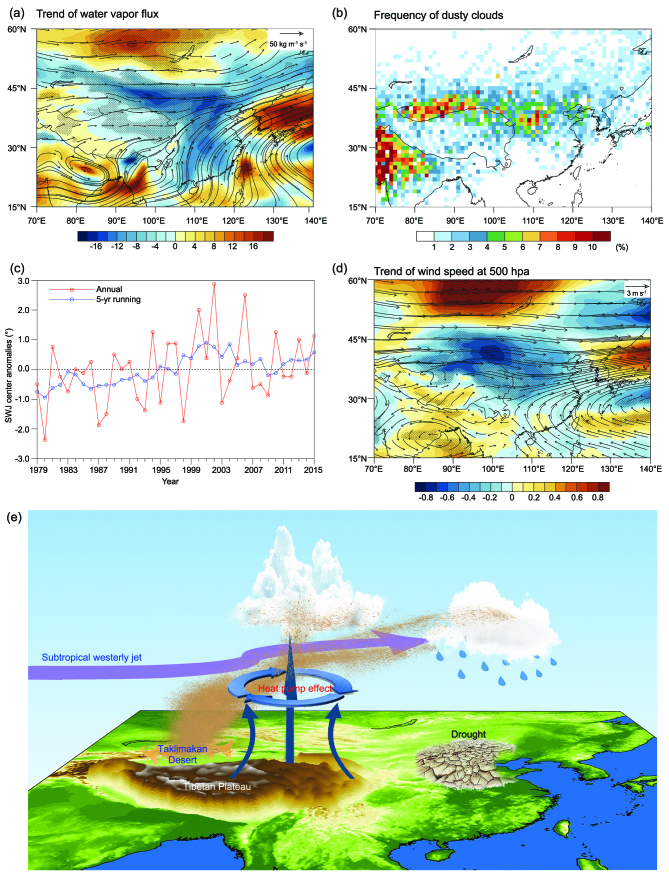
Mechanism of the TP’s attribution to northern drought. (a) Trend distribution of water-vapor flux (kg m^−^1 s^−^1) from the surface to 200 hPa (shading) and water-vapor flux vector (arrow) in July–August for the 1979–2015 period. The area enclosed by the black bold curve is the main body of the TP. The dotted symbol denotes a trend significant above the 90% confidence level. (b) The frequency distribution of dusty clouds obtained from CALIPSO and CloudSat observations during 2007–15. (c) Time series of the jet stream center's position anomalies over the region of 30–60°N, 90–110°E during 1979–2015. (d) Distribution of horizontal wind-speed trends (shading; m s^−^1 decade^−1^) and wind vectors (arrows) at 500 hPa in July-August from the ERA-interim data for the 1979–2015 period. The area enclosed by the black bold curve is the main body of the TP. The dotted symbol denotes a trend significant above the 90% confidence level. (e) Schematic of the northern drought attributed to the TP.

In summer, the TP is a significant moisture source because it produces abundant water vapor and plays an important role in supplying water vapor to the north. As indicated in Fig. 1a, since 1979, water-vapor transport has demonstrated significantly diminishing trends, and summer precipitation has presented a decreasing trend over the northern area of China [4]. Simultaneously, driven by the ‘elevated heat pump’ effect of the TP, convective clouds frequently form due to abundant water-vapor convergence over the TP, which is another kind of water resource transported to the north from the TP. In addition, the TP is located at the conjunction of several dust sources. Dust aerosols can be entrained over the TP by the convergence process [5] and transported efficiently downstream by the TP strengthening effect.

Moreover, dust aerosols can mix with clouds, forming dusty clouds (i.e. clouds in a dust-plume environment). As shown in Fig. 1b, over the boundaries of the TP, which correspond to a high cloud frequency [6], satellites frequently observe dusty clouds in summer. Dust aerosols could influence the microphysical properties of clouds by augmenting the formation and growth processes of ice crystals in the clouds. It was found that dust aerosols in convective clouds may produce a secondary indirect effect (i.e. the cloud lifetime effect) over the TP. Dust particles decrease the effective radii of ice particles in convective clouds, prolonging the life of these clouds and triggering increased cloud development over the TP [7]. Furthermore, driven by westerly circulation, these dusty clouds move eastward and merge with cloud clusters along the movement path and could thus contribute to rainfall over the northern area of China [7]. Therefore, the changes in water vapor, clouds, dust aerosols and dusty clouds transport from the TP could partially alter precipitation over the northern area of China.

Regarding the outflows of water vapor, dusty clouds, cloud condensation nuclei and ice nuclei from the TP towards the north, the intensity of the westerly wind in upper levels of the atmosphere is critical, and the position of the SWJ has a determining influence on this intensity. As indicated in Fig. 1c, the center of the SWJ indicates a significant northward shift during July and August over the region of 30°–60°N, 90°–110°E. Correspondingly, as shown in Fig. 1d, the upper-tropical wind speeds over the 30°–45°N belt, especially in the northern area of East Asia, are significantly weakened.

As illustrated above, a dynamic mechanism of the summer drought in the northern area of China is shown in Fig. 1e. As the SWJ shifted northward in the summer, particularly in July and August, during 1979–2015, the upper-level wind speed weakened significantly. Simultaneously, the wind speed in the lower tropospheric atmosphere also weakened (figures omitted). Consequently, the transport of water vapor from the TP to the north decreased and, as the outflow of dusty clouds from the TP decreased, the atmosphere over the northern area of China tended to become drier. Simultaneously, the transport of dust aerosols, which can serve as ice nuclei in clouds, from the TP to the north decreased; meanwhile, the anthropogenic aerosols, especially sulfate aerosols, demonstrated a high increasing trend over the northern area of China. In this northern area, under a relatively dry atmospheric environment and scarce dusts serving as ice nuclei in clouds, abundant anthropogenic aerosols may suppress rainfall due to a deficit in the amount of water vapor and ice nuclei. In summary, under the dynamic control of the shift in the SWJ in summer, the outflow of water vapor and clouds from the TP decreased, which is dominantly attributable to the rainfall reduction over the northern area of China. Additionally, although the role of dust aerosols in affecting the rainfall over the northern area of China is uncertain, the possible decrease in dust-aerosol transport because of the weakening of the Plateau effect on the northern drought could be non-negligible.

With global warming, northern drought may occur more frequently in China, threatening climate adaptation in this area. Thus, weather forecasting and climate prediction will face new challenges because of the resulting uncertainties. Here, we established a new position index for the SWJ that links summer precipitation over the northern area of China with the SWJ position:

(1) \begin{eqnarray*} {{\textit {Index}}_{\textit {SWJ}}} &=& {\textit {Pos}}\!\left\{ {\textit{Max}}\left[ u\!\left( 30^\circ\right.\right.\right.\nonumber\\ &&\left.\left.\left. - 60^\circ {\rm{N}},90^\circ - \! 110^\circ {\rm{E}} \right) \right] \right\}\nonumber\\ && - {\textit{Mean}}_{\textit{pos}}\end{eqnarray*}

where *Pos* denotes the latitude with the maximum wind speed over the region of 30°–60°N, 90°–110°E in the year,$\ u$ denotes the zonal mean wind speed and ${\textit {Mean}}_{\textit{pos}}$ denotes the average position of the SWJ center over the region in July and August during the investigated period. A positive ${\textit {Index}}_{\textit{SWJ}}$ value corresponds to a northward shift in the SWJ over the region, while a negative ${\textit {Index}}_{\textit{SWJ}}$ value denotes a southward shift in the SWJ. This index can be integrated into precipitation forecasting systems to improve precipitation forecasts in summer over the northern area of China (to some degree). The index is applicable for summer-precipitation forecasts over the northern area of China under a warmer climate and can aid in the prevention of flood and drought disasters.

In addition to the SWJ, associations of other branches of atmospheric circulation with the drought have been revealed, in which the changes of some circulation branches are induced by TP’s thermal effect [8]. The thermal forcing of TP in summer can contribute to strong ascent locally and the large-scale South Asian high in the upper troposphere and also be closely related to the onset and intensity of the Asian summer monsoon (ASM) [9]. Moreover, the signal of anomalous TP heating can spread outwards to a larger area. Furthermore, the contributions of the TP not only to drought in northern areas, but also to drought in other regions of China have been investigated. It was reported that weak heating by the TP and vigorous convection activities over the Philippines area are likely responsible for the extreme summer drought in southwest China. In addition, it was found that the snow in winter or spring on the TP, as the most important thermal variable, has a closely lagged impact on summer precipitation in eastern China; increased snow on the TP in the preceding winter is one of the important factors causing summer drought in northern China and southern China. In contrast, decreased winter snow on the TP usually causes the Yangtze valley to experience more severe droughts. Therefore, the influence of the TP on nearby droughts is a complex process.

In summary, the TP is considered to be attributable to the drought in northern China by different mechanisms. In general, the TP’s thermal contrast with the oceans to its south and east and snow conditions over the TP [10] can induce the weakening of the ASM, and then contribute to the drought over northern China. Importantly, the weakening trend in TP thermal forcing after the mid-1970s is considered to be associated with the weakness of the ASM and contributes to summer drought over northern China and wetness over northwestern China. Overall, the atmospheric circulation, especially the ASM and westerly jet, plays important role in the transport of water vapor, clouds, condensation nuclei and ice nuclei from the TP. So far, on the attribution of the TP to northern drought, more research has focused on the role of the TP in transporting water vapor, whereas few studies have involved the effects of clouds, condensation nuclei and ice nuclei transportation from the TP. In future, a comprehensive analysis of the impacts of atmospheric circulation, clouds, water vapor, aerosols and other aspects induced by the changes in the thermal and dynamic features of the TP under a warming climate is urgently needed.
